# Supplementary figures and images for: Systemic iron availability differentially shapes tumor and brain iron handling in a sex-dependent manner in glioblastoma
Source: PLoS One. 2026 Apr 20;21(4):e0347520. doi: 10.1371/journal.pone.0347520 (PMC13095122; doi:10.1371/journal.pone.0347520)

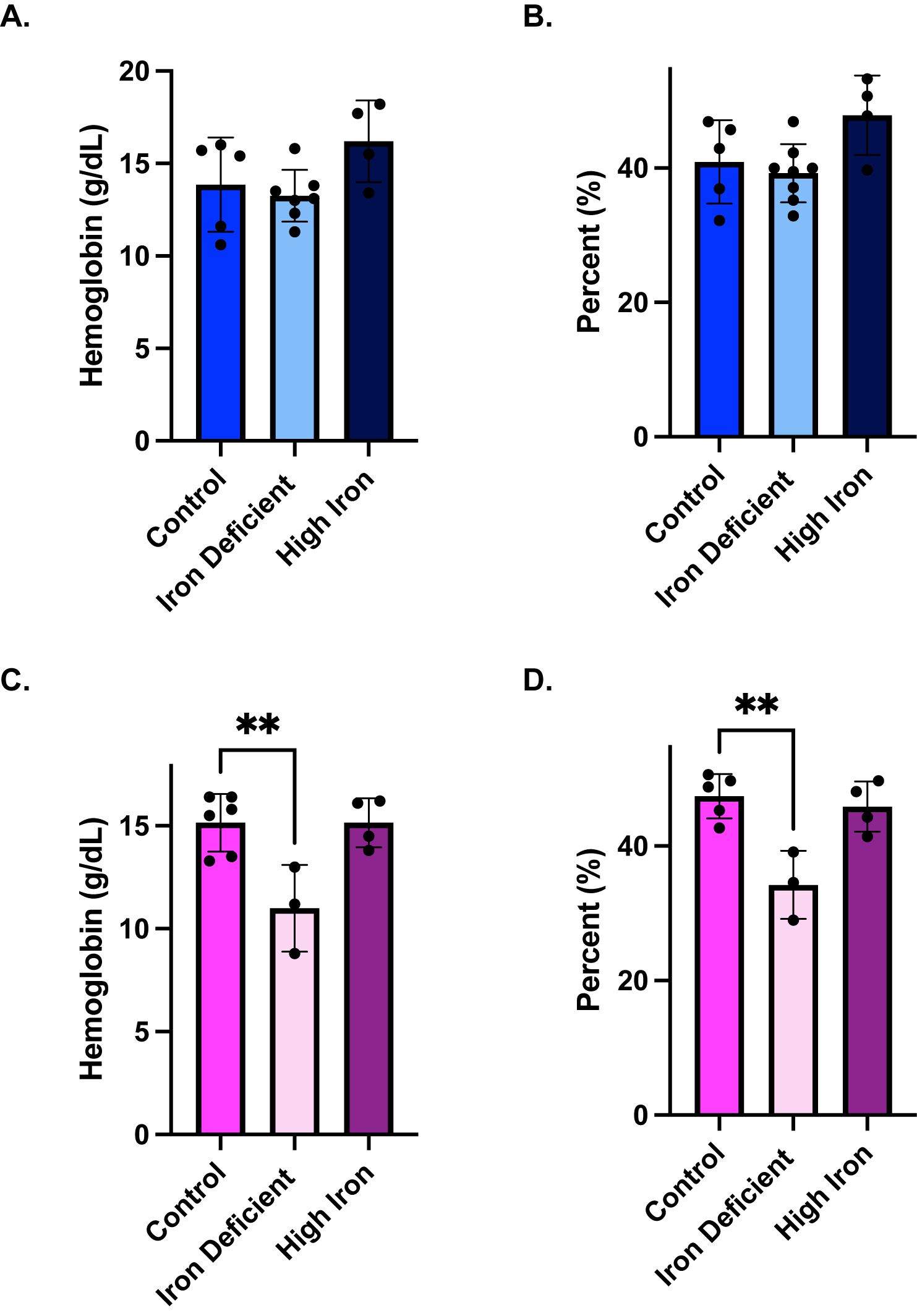

Supplement: S1 Fig — A-B) Mean hemoglobin and hematocrit measures in males (±SD): At the experimental endpoint, there were no significant differences in hemoglobin between control and iron deficiency anemia (IDA) mice (p = 0.83) or high iron mice (p = 0.18). There were also no significant differences in hematocrit between control and IDA mice (p = 0.80) or high iron mice (p = 0.12). C-D) Mean hemoglobin and hematocrit measures in females (±SD): At the experimental endpoint, IDA females exhibited significantly reduced hemoglobin (**p = 0.006) and hematocrit (**p = 0.002) compared to controls. There were no significant differences in the high iron group compared to controls for hemoglobin (p > 0.99) or hematocrit (p = 0.79). (TIF) [file pone.0347520.s001.tif]

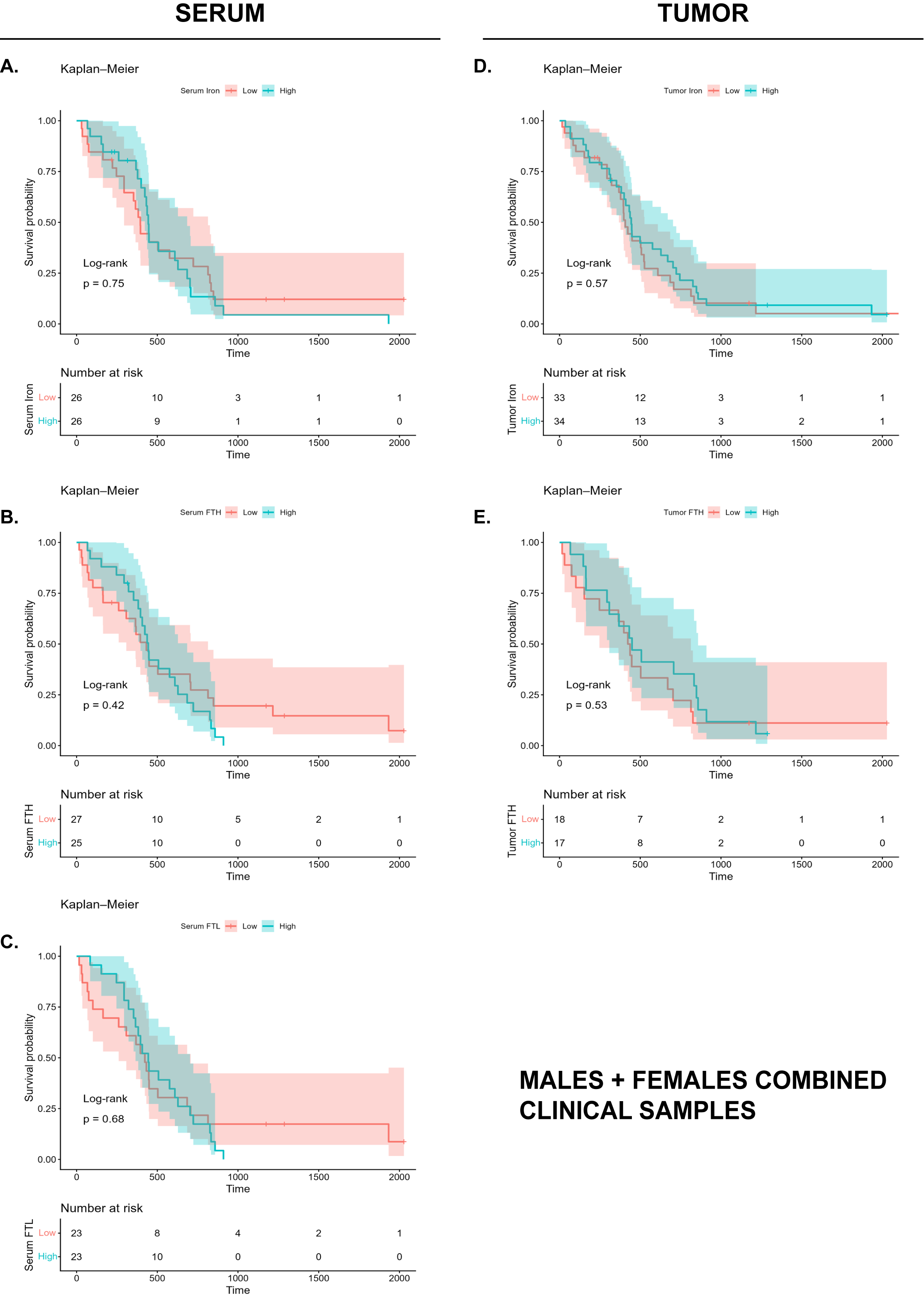

Supplement: S2 Fig — A) Kaplan-Meier survival analysis of clinical samples of high versus low serum iron. No significant differences in survival were observed (p = 0.75) B) Kaplan-Meier survival analysis of high versus low serum ferritin heavy chain (FTH). No significant differences in survival were observed (p = 0.42). C) Kaplan-Meier survival analysis of high versus low serum ferritin light chain (FTL). No significant differences in survival were observed (p = 0.68). D) Kaplan-Meier survival analysis of high versus low tumor iron. No significant differences in survival were observed (p = 0.57) E) Kaplan-Meier survival analysis of high versus low tumor FTH. No significant differences in survival were observed (p = 0.53). (TIF) [file pone.0347520.s002.tif]

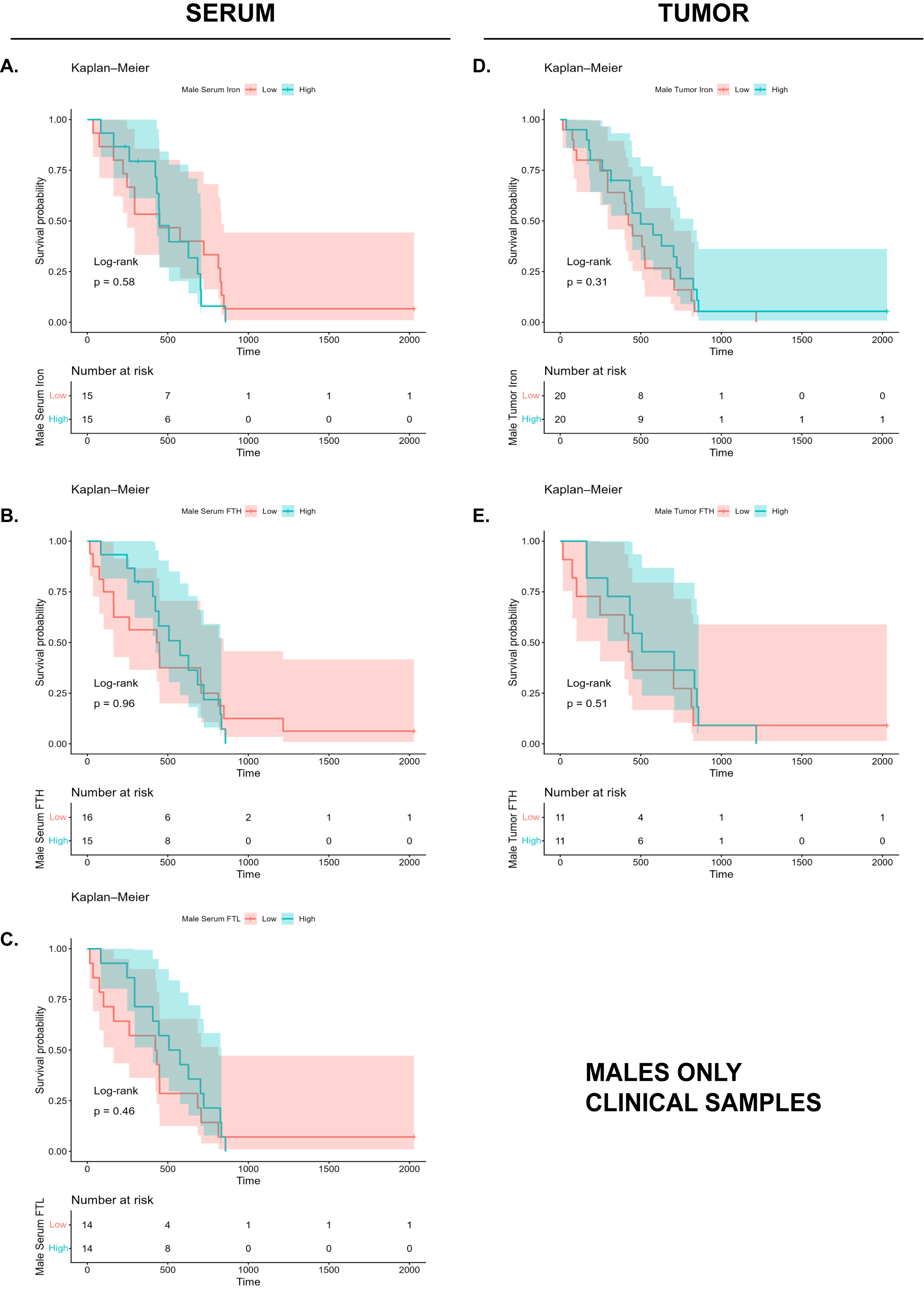

Supplement: S3 Fig — A) Kaplan-Meier survival analysis of male clinical samples of high versus low serum iron. No significant differences in survival were observed (p = 0.75). B) Kaplan-Meier survival analysis of high versus low serum ferritin heavy chain (FTH). No significant differences in survival were observed (p = 0.96). C) Kaplan-Meier survival analysis of high versus low serum ferritin light chain (FTL). No significant differences in survival were observed (p = 0.46). D) Kaplan-Meier survival analysis of high versus low tumor iron. No significant differences in survival were observed (p = 0.31) E) Kaplan-Meier survival analysis of high versus low tumor FTH. No significant differences in survival were observed (p = 0.51). (TIF) [file pone.0347520.s003.tif]

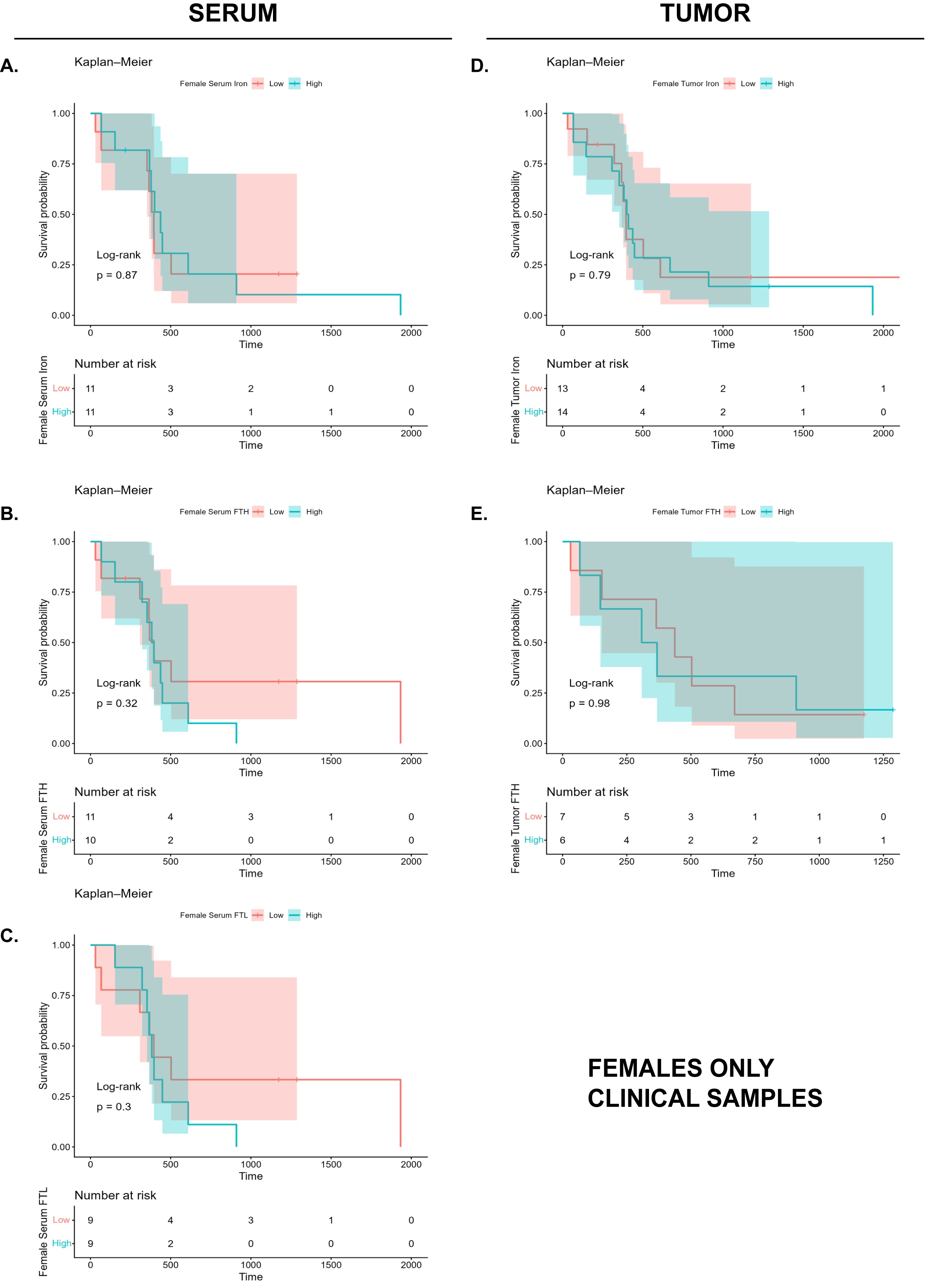

Supplement: S4 Fig — A) Kaplan-Meier survival analysis of female clinical samples of high versus low serum iron. No significant differences in survival were observed (p = 0.87). B) Kaplan-Meier survival analysis of high versus low serum ferritin heavy chain (FTH). No significant differences in survival were observed (p = 0.32). C) Kaplan-Meier survival analysis of high versus low serum ferritin light chain (FTL). No significant differences in survival were observed (p = 0.30). D) Kaplan-Meier survival analysis of high versus low tumor iron. No significant differences in survival were observed (p = 0.79) E) Kaplan-Meier survival analysis of high versus low tumor FTH. No significant differences in survival were observed (p = 0.98). (TIF) [file pone.0347520.s004.tif]
