# Supplementary material for: Systemic iron availability differentially shapes tumor and brain iron handling in a sex-dependent manner in glioblastoma
Source: PLoS One. 2026 Apr 20;21(4):e0347520. doi: 10.1371/journal.pone.0347520 (PMC13095122; doi:10.1371/journal.pone.0347520)

Systemic iron availability differentially shapes tumor and brain iron handling in a sexdependent manner in glioblastoma

Emily Tufano[1], Kondaiah Palsa[1], Rebecka O. Serpa[1,2,3], Timothy B. Helmuth[1], Gabriela Remit-Berthet[1], Sara Mills-Huffnagle[2,3], Mathias Kant[1], Aurosman Sahu[1], & James R. Connor[1,2,3*]

[1] Department of Neurosurgery, The Pennsylvania State University College of Medicine, Hershey,

PA, United States of America

[2] Department of Neuroscience and Experimental Therapeutics, The Pennsylvania State University College of Medicine, Hershey PA, United States of America

[3] Penn State Neuroscience Institute, The Pennsylvania State University College of Medicine, Hershey, PA, United States of America

***Corresponding Author:**

Email: jconnor@pennstatehealth.psu.edu (JRC)

Figure 7: Transferrin Receptor (TfR1) and Ferritin Light Chain (FTL) Images

Uncropped Western Blot Data:

Figure 7A: Female TfR1

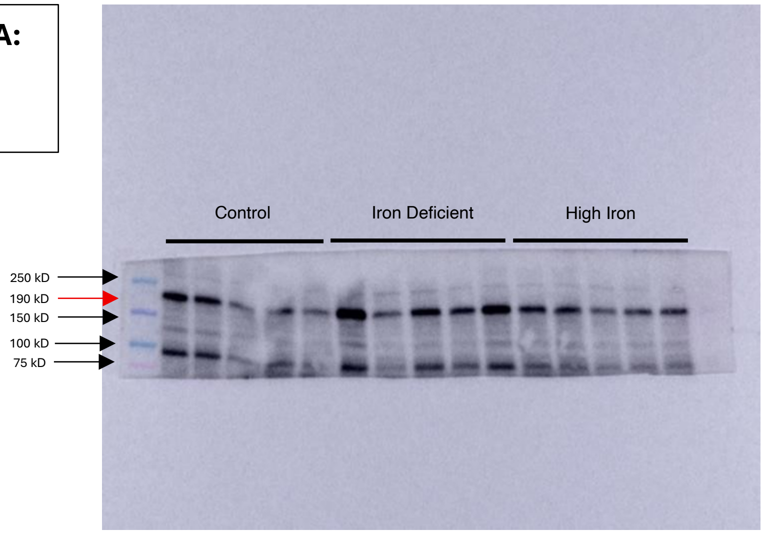

Figure 7A: Female ß-Actin

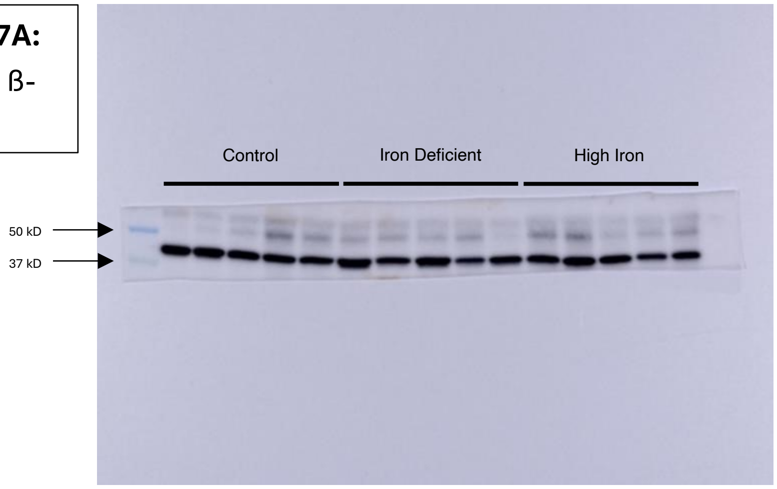

Figure 7B:
Male TfR1

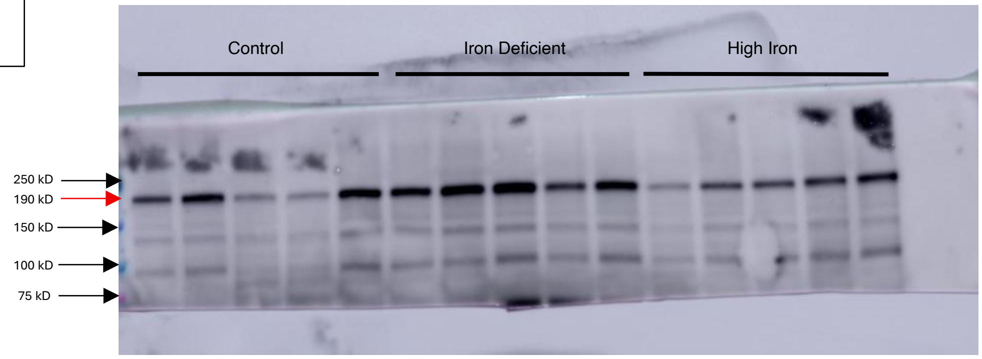

Figure 7B:
Male ß-Actin

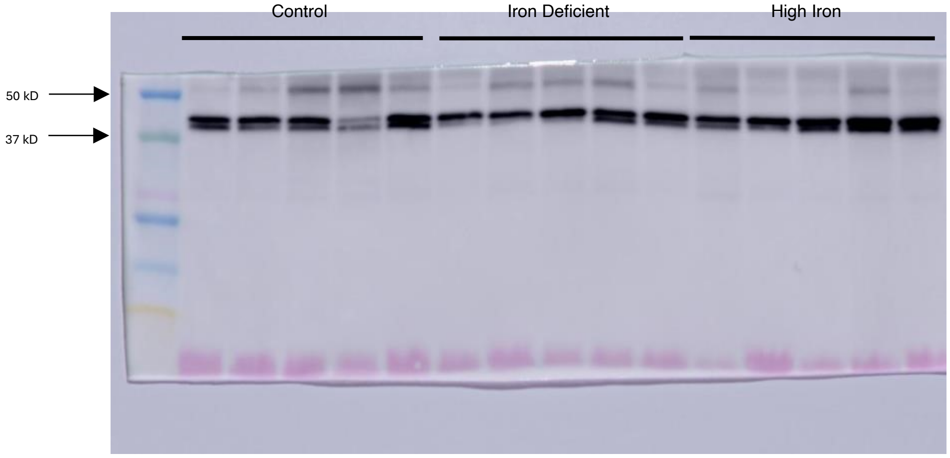

Figure 7C:
Female FTL

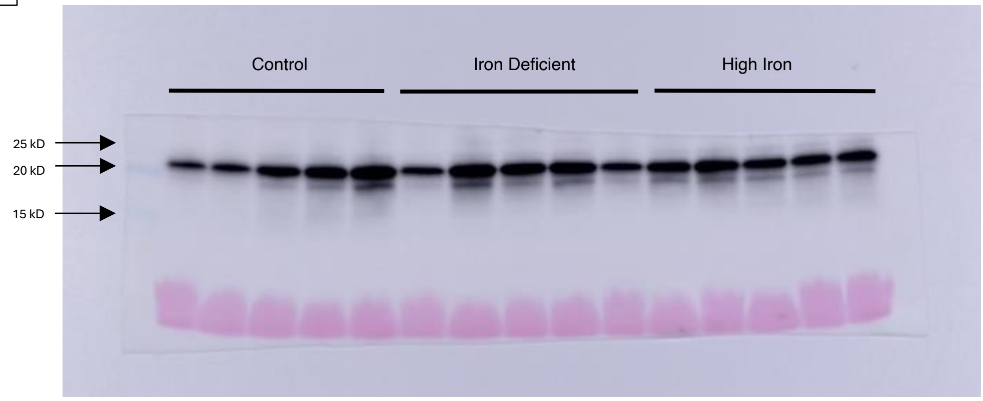

Figure 7C:
Female ß-Actin

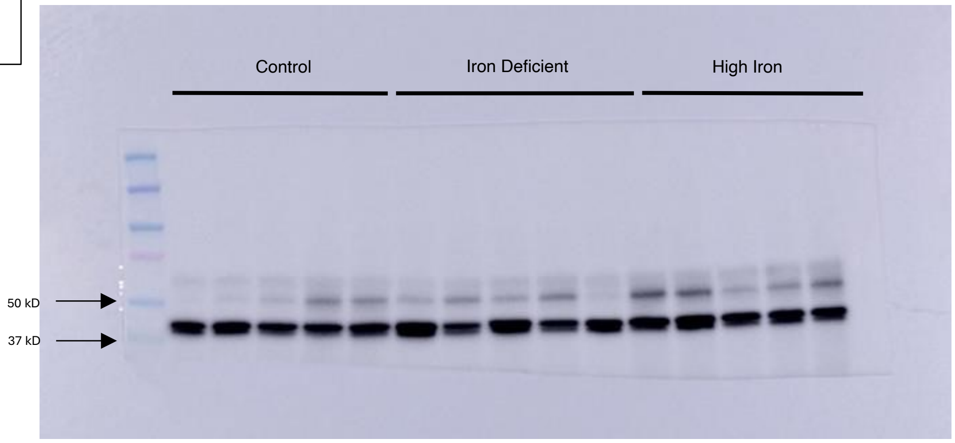

Figure 7D:
Male FTL

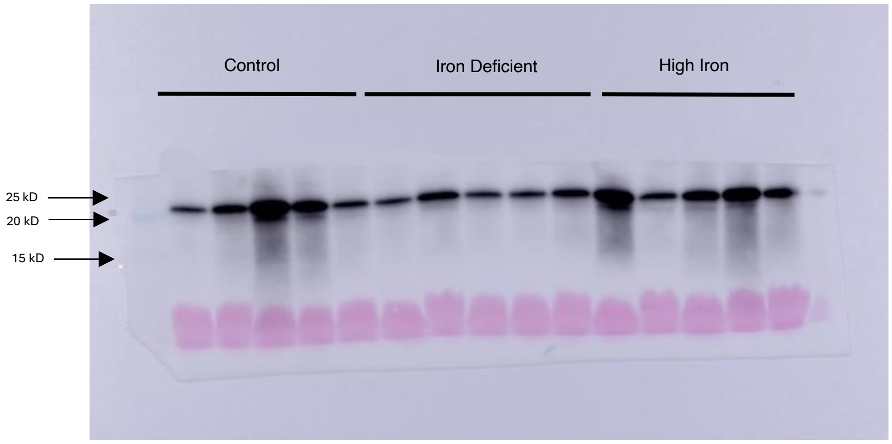

Figure 7D:
Male ß-Actin

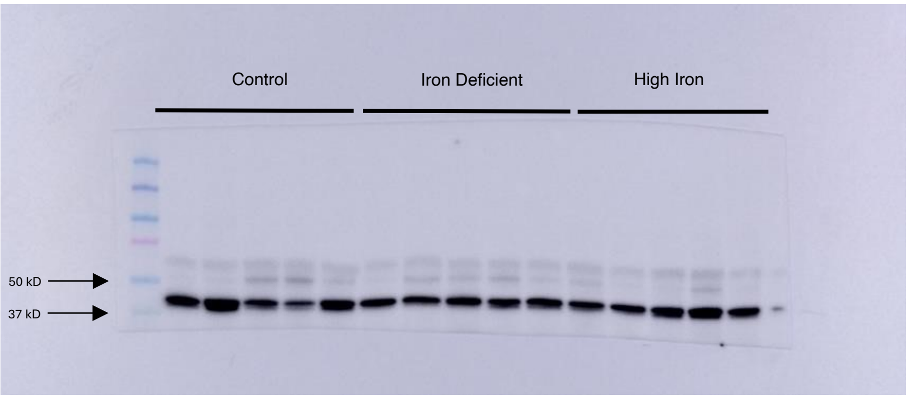

Supplement: S5 Fig — Full uncropped images of transferrin receptor (TfR1, approx. 190kD), ferritin light chain (FTL, approx. 20kD), and corresponding β-actin (approx. 42kD) in tumor lysates in males and females. Lanes 1–5: Control; Lanes 6–10: Iron Deficient; Lanes 7–15: High Iron. Loading schemes were kept consistent for all gels. (PDF) [file pone.0347520.s005.pdf]
