# Supplementary material for: Systemic iron availability differentially shapes tumor and brain iron handling in a sex-dependent manner in glioblastoma
Source: PLoS One. 2026 Apr 20;21(4):e0347520. doi: 10.1371/journal.pone.0347520 (PMC13095122; doi:10.1371/journal.pone.0347520)
Supplement: S2 Table — Multivariate cox proportional hazard regression of clinical outcomes for all factors. Hazard ratios, 95% confidence intervals, and associated p-values are reported for all factors. The regression was performed controlling for relevant clinical covariates such as patient sex, age at diagnosis, white blood cell count, extent of tumor resection, receipt of chemotherapy, dexamethasone, and/or radiation. (DOCX) [file pone.0347520.s007.docx]

Cox-model WITH Resection Volume & WBC

|  | Hazard Ratio | 95% Confidence Interval | P Value |
| --- | --- | --- | --- |
| Serum Iron High vs Low | 0.72 | (0.36 – 1.46) | 0.366 |
| Serum Iron Males High vs Low | 0.84 | (0.29 – 2.44) | 0.745 |
| Serum Iron Females High vs Low | 0.797 | (0.21 – 3.31) | 0.797 |
| Serum FTH High vs Low | 0.76 | (0.36 – 1.61) | 0.481 |
| Serum FTH Males High vs Low | 0.68 | (0.22 – 2.07) | 0.498 |
| Serum FTH Females High vs Low | 2.35 | (0.19 – 29.00) | 0.505 |
| Serum FTL High vs Low | 0.82 | (0.40 – 1.68) | 0.58 |
| Serum FTL Males High vs Low | 0.78 | (0.22 – 2.70) | 0.69 |
| Serum FTL Females High vs Low | 15.75 | (0.46 – 538.2) | 0.126 |
| Tumor Iron High vs Low | 0.98 | (0.55 – 1.77) | 0.956 |
| Tumor Iron Males High vs Low | 0.56 | (0.16 – 1.90) | 0.349 |
| Tumor Iron Females High vs Low | 0.90 | (0.29 – 2.77) | 0.85 |
| Tumor FTH High vs Low | 0.65 | (0.29 – 1.45) | 0.295 |
| Tumor FTH Males High vs Low | 0.52 | (0.18 – 1.53) | 0.236 |
| Tumor FTH Females High vs Low | 2.25 | (0.34 – 15.02) | 0.404 |
