# Supplementary material for: Systemic iron availability differentially shapes tumor and brain iron handling in a sex-dependent manner in glioblastoma
Source: PLoS One. 2026 Apr 20;21(4):e0347520. doi: 10.1371/journal.pone.0347520 (PMC13095122; doi:10.1371/journal.pone.0347520)
Supplement: Attachment — Submitted filename: Responses to Reviewers - E Tufano - 02-24-26.pdf [file pone.0347520.s009.pdf]

PennState College of Medicine

Department of Neurosurgery
Mail Code H110
500 University Drive
Hershey, PA 17033
717-531-4541 Tel
717-531-0091 Fax

Kevin M. Cockroft, MD, MSc, FAANS, FACS, FAHA
Professor & Chair, Neurosurgery
Neurovascular Neurosurgery

Dawit G. Aregawi, MD
Medical Neuro-Oncology

Gregory D. Arnone, MD
Neurosurgery at Holy Spirit & Lancaster

Ephraim W. Church, MD, FAANS, FACS
Neurovascular Neurosurgery

James R. Connor, PhD
Neuroscience Research

Mark S. Dias, MD, FAANS, FAAP
Pediatric Neurosurgery

Robert E. Harbaugh, MD, FAANS, FACS, FAHA
Senior Advisor

Mark R. Iantosca, MD, FAANS
Pediatric Neurosurgery

Diana H. Jho, MD
Neurosurgery at Holy Spirit

Rong Jin, MD, PhD
Neuroscience Research

John P. Kelleher, MD, FAANS
Spine Surgery

Guohong Li, MD, PhD
Neuroscience Research

S. Alireza Mansouri, MD, MSc, FRCSC
Neurosurgery at Holy Spirit & Lancaster

Michael M. McDowell, MD
Pediatric Neurosurgery

James McInerney, MD, FAANS, FACS
Functional & Radiosurgery

Paul B. Nelson, MD
Neurosurgery at University Park

Inan Olmez, MD
Medical Neuro-Oncology & Neuroscience Research

Brent R. O'Neill, MD
Pediatric Neurosurgery

Haejoe Park, MD, FAANS
Neurocritical Care and Neurotrauma

Elizabeth A. Proctor, PhD
Neuroscience Research

G. Timothy Reiter, MD, FAANS
Spine Surgery

Michael D. Sather, MD, FAANS
Epilepsy Surgery

Scott D. Simon, MD, FAANS
Neurovascular Neurosurgery

John P. Weaver, MD
Neurosurgery at Saint Joseph & Lancaster

David A. Wilkinson, MD
Neurovascular Neurosurgery

Qing X. Yang, PhD
Neuroscience Research

Brad E. Zacharia, MD, MS, FAANS
Neurosurgical-Oncology and Skull Base Surgery

J. Christopher Zacko, MD, MS, FAANS
Neurocritical Care and Neurotrauma

Joint Faculty

Scott B. Armen, MD
Arthur Berg, PhD
Gong Chen, PhD
Patrick J. Drew, PhD
Elana Farace, PhD
Andrew Geronimo, PhD
Bruce Gluckman, PhD
Neerav Goyal, MD
S. William Hazard, MD
Ibrahim Ozbolat, PhD
Huang Xuemei, PhD
Paul Kalapos, MD, FRCP
Melissa Linskey, MD
John F. Radtka, MD
Thomas D. Samson, MD
Semyon M. Slobounov, PhD
Charles Specht, PhD
Vijay Varadan, PhD

An Equal Opportunity Employer

February 18th, 2026
PLOS One

Subject: Resubmission of Manuscript, "Systemic Iron Availability Differentially Shapes Tumor and Brain Iron Handling in Glioblastoma" [PONE-D-25-68105]

Dear Dr. Kostas Pantopoulos and Editorial Team,

Thank you for the opportunity to review and resubmit our Formerly: "Systemic iron availability differentially shapes tumor and brain iron handling in glioblastoma". We have revised the title to "Systemic Iron Availability Differentially Shapes Tumor and Brain Iron Handling in a Sex-Dependent Manner in Glioblastoma." We sincerely thank the reviewers for their thoughtful and constructive feedback, which has greatly improved the overall quality of our manuscript.

Below, we provide a detailed response to each comment. For ease of review, the reviewers' comments are presented in bold, followed by our responses in regular text. Specific revisions are indicated by yellow highlighting within the response document where the changes are brief. For more extensive revisions, we direct to the reviewer to the relevant section and provide a concise summary. All corresponding changes are tracking in red in the revised manuscript. Additionally, we have addressed the additional requirements of the journal, including removal of the funding statement, updating the manuscript style, and attaching uncropped and unadjusted blot images as a Supporting Information file.

We are very grateful for the reviewers' time and insights, and we expect the revisions have addressed all concerns and that the manuscript meets the journal's expectations.

Sincerely,

James R. Connor, Ph.D.
University Distinguished Professor
Vice-Chair of Neurosurgery
Director, G.M. Leader Family Laboratory
Penn State University College of Medicine

REFEREE 1:

The manuscript by Tufano et al describes a study analysing the role of iron in glioblastoma, using a mouse model and a cohort of patient samples. It is a careful study with complex, and some robust findings in the animal model, but clinical data is overinterpreted.
Below are detailed comments and questions on the manuscript.

1. **Is there an overlap of clinical GBM samples/data with previous study (Shenoy Transl Oncol 2025)?**

A total of 40 samples overlapped between this study and Shenoy et al. (2025). All processing and analyses in the present manuscript were performed by the investigators on this manuscript using separate aliquots of serum and tumor tissue, following the methods described in Shenoy et al. (2025). Notably, the Shenoy et al. (2025) paper reports including only 21 of these overlapping samples into their survival analysis, but the specific subset is not specified. We report using 52 samples for our corresponding serum analysis.
We discuss discrepancies in these results in response to reviewer's comment 29 and in the discussion of the manuscript (please see comment #29)

2. **Do mice have similar sex differences as humans with regard to iron levels and metabolism? Is this a relevant model?**

The reviewer raises a good point on the relevance of the model, and we acknowledge the limitations of our approach. However, there is considerable and substantial information on the relevance of mouse models in iron biology in a recent review entitled: "Regulation of iron homeostasis: Lessons from mouse models," (PMID: 32792212) though sex differences are not mentioned.

We have added a summary sentence on the relevance of our model in the introduction. In this section and its associated references, are several key points regarding model relevance: In human GBM, males exhibit significantly higher binding of radiolabeled FTH compared to females under baseline conditions. Consistent with this, we observe higher tumor FTH levels in male mice compared with females in the control diet groups, though uptake was not directly measured. Additionally, our preclinical survival findings parallel those reported in Shenoy et al. (2024), including sex-dependent relationships between anemia and survival. In terms of general iron biology, as the reviewer points out in #17 and #18, there is an elevation of tumor iron compared to contralateral brain tissue in our preclinical model. This finding has been cited in the literature for human GBM samples, reporting elevated iron uptake in GBM tissues compared to surrounding brain tissue, likely due to the elevated transferrin receptor.

The revised manuscript now reads:

Introduction: This immune competent model recapitulates key features of GBM, and in our study this model was able to reproduce sex-dependent iron and ferritin patterns observed in patients, supporting its translational relevance [15-17]. To complement these studies, we analyzed human GBM tumor and serum samples for circulating iron and ferritin levels.

3. **Overall, the order of figures is confusing and could be made more logical.**

In response to the reviewer, we have updated the order of the figures and results in a way that we view as more logical. The story is now described from the iron perspective (brain iron (Figure 4), tumor iron (Figure 5), and serum iron (Figure 6), followed by their related iron proteins (tumor iron proteins (Figure 7) and serum iron proteins (Figure 8 and 9).
Additionally, we have changed the order of the figures within Figure 7 to be consistent with Figures 5-6 and 8-9.

4. **Human ethics Define 'honest broker' Animal ethics Acceptable**

Further clarification regarding the honest broker system has been added to the Methods section, The Penn State Biorepository staff member who coordinated sample dissemination and patient data collection is a co-author on this manuscript but was not involved in patient sample analysis. The investigators responsible for the experimental analyses were not, and remain not, privy to identifiable patient information, ensuring appropriate separation between clinical data handling and research activities.

The revised manuscript now reads:
Methods Section 2.10: Clinical information for each patient was extracted from the electronic medical record by a neutral system (honest broker) in accordance with the Biorepository protocol to further protect privacy by separating patient identifiers from samples and results.

5. **Title: Should the title reflect the importance of sex differences?**

We agree with this point that the reviewer has raised, and have edited the title in response:
Systemic iron availability differentially shapes brain and tumor iron handling in a sex-dependent manner in GBM

6. **Line 39 Overinterpretation – there is no evidence from clinical data for any association with patient survival**

We accept this point from the reviewer and have updated the manuscript to prevent overinterpretation of the clinical results, while still describing the importance.

Abstract: In clinical GBM samples, we observed similar survival trends across varying iron and ferritin levels, suggesting potential translational relevance.

7. **Line 56 Oxygen production?**

We have updated the manuscript to reflect the correct wording.

Introduction: Iron is an essential element that is required for many physiological processes, including oxygen transport, DNA replication and repair, and neurotransmitter synthesis (5).

8. **Line 112 Method clarification needed: What diet was the high-iron group on prior to tumour implant? Were the control and IDA groups maintained on their respective diets (since PND 21) post tumour implant?**

The high-iron group was on control diet prior to tumor implant. Control and IDA groups were maintained on their respective diets throughout the study. Clarification has been added to the methods section:

Methods Section 2.1: At the time of tumor implantation, iron-deficient mice continued on iron deficient diets, whereas control diet mice were randomized to either remain on control diets or transition to high iron diets. Diets were maintained for the remainder of the experiment (until the primary endpoint, about 17-37 days post injection), resulting in three experimental groups: control (female n = 8, male n = 9), iron deficient (female n = 8, male n = 9), and high iron (female n = 8, male n= 8; 350 mg/kg Fe, Global rodent diet, 2018 Teklad 18% Protein Rodent Diets, Cat. #NC0035505) diets.

9. **Line 175 A loss of 9/50 animals due to monitoring timing seems a waste.**

We appreciate the reviewer's concern regarding animal loss during monitoring. Importantly, animal welfare was prioritized throughout the study. GBM intracranial models are characterized by rapid and unpredictable neurological decline, which can occur in a very short time window during late-stage disease. Although animals were monitored closely in accordance with the IACUC guidelines, several animals experienced such deterioration outside of the scheduled monitoring periods. Animals that progressed rapidly or that could not be reliably perfused were excluded from tissue analyses and accounted for in the reported 9/50. This exclusion was necessary to limit the post-mortem interval. Additionally, this precaution prevented compromised integrity in downstream tissue iron levels from blood pooling or post-mortem iron release.

10. **Line 193 Western blot data for FTH1 not shown, why listed here?**

This was an error that has been removed from the Methods Section.

11. **Line 228, 246 No data on multivariate cox proportional hazards regression is presented. And sentence repeated.**

In response to the reviewers, we have replaced the Kaplan-Meier curves in the main figures with the cox proportional hazards regression. The results/figure legends have been updated to represent these results. The Kaplan-Meier curves have been relocated to Supplementary Figures 2-4. We have also deleted the repeat sentence.

12. **Fig 3 Why was day 10 chosen? The majority of male mice on control diet did not have measurable tumours. And the majority of female mice on iron deficiency diet similarly showed no tumour. Legend should specify 10 days. No significant difference in survival.**

We agree with the reviewer on this point. Day 10 post-tumor injection was selected based on prior literature using this model, which demonstrates reliable tumor establishment ~2mm by 10 days. We intentionally chose an earlier time point both to confirm tumor growth and uptake dynamics prior to the onset of rapid tumor expansion and endpoint-related changes.

As the reviewer notes, a substantial portion of male mice on control diet and female mice on IDA diet did not yet exhibit measurable tumors at day 10. We interpret this as biologically informative, reflecting delayed early growth in these groups. Importantly, this early imaging time point was not intended to predict survival outcome, but the trending relationship between the two was worth mentioning in the manuscript.

The figure legend has been revised to explicitly state that tumor volume was assessed by MRI at 10 days post injection. A brief interpretation of these results has been added to the discussion section:

<u>Discussion:</u> Notably, earlier tumor volume measurement (10 days post injection) in this model provided insight into tumor establishment and iron-dependent growth dynamics. At this stage, several male control and female IDA mice did not yet exhibit measurable tumors, suggesting delayed tumor initiation or early growth restraint in these groups that may have impacted downstream survival outcomes.

13. **Fig 4 and 5 There is no evidence from mice that high iron diet resulted in different tumour or brain tissue levels compared to controls. The period of dosing may have been too short. Figure 7 shows blood data – suggest moving closer.**

We agree with the reviewer that exposure to high dietary iron did not result in a significant increase in tumor or brain tissue in this model. This finding suggests, and further supports our conclusion, that iron is tightly regulated at the CNS and does not necessarily reflect systemic iron. As the reviewer notes, it is possible that a longer period of exposure may be required to overcome this regulation and induce detectable changes in our outcomes. Our primary rationale for this regimen was to enhance clinical translatability: In the clinical setting, iron supplementation (or dietary iron changes) would almost exclusively occur after diagnosis or in response to anemia identified during treatment rather than prolonged or prior exposure. Therefore, initiating high iron dosing at tumor injection models the earliest realistic intervention point following diagnosis.

While the serum iron data is not statistically significant between controls and high iron groups, high iron males had 68% increase in serum iron compared to controls, while females exhibited a 67% increase. As mentioned, these changes were not complimented by increases in tumor or contralateral hemisphere iron, supporting the conclusion that there are unknown regulatory mechanisms limiting iron uptake at the tissue level despite altered circulating iron.

To improve clarity and better integrate these findings, we have moved the serum iron data to Figure 6, directly following contralateral and tumor iron content.

14. **Line 346 A rather optimistic heading as only male mice showed this association; female mice and human patients did not.**

We have edited the section title to more accurately reflect these associations:

Section 3.4: Sex-dependent tumor iron content is associated with survival

15. **Line 363 There is no trend in clinical data (p=0.31, 0.57 and 0.79).**

We agree with the reviewer and have changed the wording to reflect similarities in survival directionality between clinical and preclinical models, while emphasizing that the clinical findings are not statistically significant. These changes also reflect the cox hazards regression.

The revised manuscript now reads:
Section 3.4: When examining clinical data, there is a modest separation in survival curves observed in males, with higher tumor iron associated with slightly longer survival (HR = 0.56 (0.16 – 1.90), p = 0.35), whereas no comparable separation was evident in females (HR = 0.90 (0.29 – 2.77), p = 0.85) or in the combined cohort (HR = 0.98 (0.55 – 1.77), p = 0.96) (Figs 5I-K). Despite the lack of statistical significance in both males and females, the directionality of this pattern was consistent with those observed in the preclinical model.

16. **Would it be worth investigating the amount of iron the tumour was able to accumulate in relation to (surrounding/contralateral) normal brain?**

We agree with the reviewer that this is an interesting observation. Notably, the tumor has 2x the iron than that of the contralateral hemisphere, which supports the translatability discussed under point #2.

In the present study, contralateral hemispheric iron was used as more of a control to assess brain iron regulation and to evaluate sex-dependent differences at the CNS level. Future studies incorporating spatial resolution or cell-specific changes in iron uptake will be valuable for defining how systemic iron alters the tumor and contralateral hemispheric iron relationship.

17. **Tumour contains notably more iron than normal brain. Discuss possible reasons.**

This is an important observation by the reviewer and highlights a key translatable component of our model. GBM tumors are highly vascularized, which likely contributes to enhanced iron content specifically through blood components. In addition, GBM cells exhibited elevated transferrin receptor expression and iron demand to support their proliferative and metabolic needs, further contributing to increased iron uptake when compared to surrounding brain tissue. This phenomenon is discussed briefly in the introduction section of the manuscript.

18. **Different parts of the brain accumulate different levels of iron; was this considered?**

The reviewer makes a good point. In this study, tumors were consistently implanted in the same cortical region, which allowed us to minimize variability related to regional differences in brain iron content. While these regional differences the reviewer mentions are an important area for future investigation, mapping of iron distribution across different brain regions was beyond the scope of our present study.

19. **Fig 6 The writing on several graphs is too small to decipher (ie p-values).**

Figure 6 has been revised to increase font size 3 points for all p-values

20. **Fig 7 No clear difference between control and high iron diet measures.**

We agree with the reviewer and have addressed this in point 13. We have also revised the text to explicitly acknowledge the lack of statistical significance yet large change in serum iron values. As previously mentioned, despite these changes in serum iron between control and high iron diet, brain and tumor iron measurements remained unchanged.
Discussion: While the animals were anemic based on hemoglobin and hematocrit measures, serum iron did not differ significantly between control and iron deficient groups or control and high iron groups. It should be noted, however, that high iron males had a 68% increase in serum iron compared to controls, while females exhibited a 67% increase. These increases were not complimented by changes in survival or iron levels in the

tumor or brain tissue, further suggesting that systemic iron availability does not directly translate to tissue-specific iron levels.

21. Line 457 There is no trend in clinical data (p=0.75, 0.58, 0.87).

We have updated the manuscript in response to the reviewer to accurately reflect the statistical outcomes.

Section 3.6: However, when males and females were evaluated separately, there were opposing relationships. Male mice with high serum iron had a slight survival advantage over males with low serum iron whereas high serum iron was slightly disadvantageous in females (25 vs 27 days, p = 0.09) (Figs 6F-G), but these trends were not significant in the preclinical model. Similarly, no evidence of a significant survival association with serum iron was observed in the clinical cohort for the combined cohort (HR = 0.72 (0.36 – 1.46), p = 0.37) or males or females (males: HR = 0.84 (0.29 – 2.44), p = 0.75; females: HR = 0.84 (0.21 – 3.31), p = 0.80) (Figs 6H-J).

22. Clarification: does FTL refer to tissue levels and L-ferritin to serum levels? Similarly, for FTH and H-ferritin?

We appreciate the reviewer pointing out this inconsistency. Originally, it was labeled this way per the ELISA kit title, but they are the same and do not reflect tissue specific ferritin. We have updated figures 6-9 (From H/L-Ferritin to FTH/L) to remain consistent.

23. Fig 8 Ensure graph orientation (x-y) to be the same for B, C and D. p-values for F and G moved off the graph.

We appreciate the reviewer's attention to detail on the figures, and for catching these errors. Figure 8 and 9 have been edited with the correct orientation.

24. Fig 9 Ensure graph orientation to be the same for B, C and D.

Please see response to point #22 above.

25. Line 503 No evidence that low serum FTL provided advantage to females.

We have edited our wording in response to the reviewer to reflect the cox hazards regression.

The revised manuscript now reads:
Section 3.7: In the clinical samples, serum FTL did not provide a survival advantage in either direction for males (HR = 0.78 (0.22 – 2.70), p = 0.69). However, low serum FTL, similar to the preclinical model, provided a survival benefit for females, though not statistically significant (HR = 15.75 (0.46 – 538.20), p = 0.13) (Figs 9H-J).

26. Fig S1 not mentioned in Results.

Supplementary figure 1 was added to the results with pre-tumor measurements. Figure legends for all supplementary material can be found in the "Supporting Information" section.
The revised manuscript now reads:
Section 3.1: Hemoglobin and hematocrit were also measured at the humane endpoint. Interestingly, IDA females had significantly lower hemoglobin compared to control mice (11.0 ± 2.107 vs 15.15 ± 1.401), while IDA males did not (13.26 ± 1.394 vs 13.86 ± 2.553) (S1 Fig A,C)). This relationship also held true for hematocrit, where IDA females were significantly lower than controls (34.23 ± 5.060 vs 47.42 ± 3.316) and IDA males were not (39.24 ± 4.315 vs 40.92 ± 6.219) (S1 Fig B,D).

27. Line 551 Not convinced that 'both models demonstrated sex-dependent patterns'

In light of the reviewer's comment, we have updated the Discussion section to reflect the data in a more convincing manner:

Discussion: Despite these differences, the preclinical model demonstrated clear sex-dependent effects, with the clinical data showing parallel directionality, highlighting the benefit of integrating both models to contextualize iron regulation in GBM.

28. Line 566 'consistent with prior literature' but no references given

We have updated our references in the discussion to properly cite this finding.

29. Their previous paper (Shenoy Transl Oncol 2025) showed a robust relationship between serum iron and survival outcome in patients with GBM, which was not supported in this bigger patient cohort. Discuss

In our prior study (Shenoy et al., Transl Oncol, 2025), we observed a significant association between serum iron and survival in GBM. However, this analysis was performed with a substantially smaller cohort of 21 matched patients, which required sample processing and further stratification into high- and low- iron groups consisting of 7 and 4 patients, respectively. The current study leverages a larger cohort of 51 matched patients, providing greater statistical power and more representation of patient variability. Additionally, we performed our analysis controlling for the same parameters as the Shenoy (2025) paper. Moreover, our study also controlled for dexamethasone. We included this parameter because dexamethasone can cause changes in peripheral white blood cell counts and has been shown to reduce survival in GBM patients.

We further emphasize a strong point raised from Shenoy et al., which is that clinical data trial interrogating the benefit of increased serum iron is required to draw conclusions on the role of iron in GBM. In this context, our preclinical model provides an important and complementary method to control variability through standardized diets, tumor initiation, treatment (or lack thereof in our case), etc. While the clinical analyses did not reach statistical significance, we observed potential translatable patterns with our preclinical model that warrant investigation in larger patient cohorts.

We have raised these points in the discussion section as follows:

Discussion: In our current analysis of clinical GBM samples, no statistically significant associations were observed between high and low iron or ferritin levels within the serum, which contrasts previous reports that have identified a robust relationship between serum iron and survival in a smaller patient cohort [30]. Many studies examining the role of anemia in GBM produce variable results, highlighting the complexity of interpreting clinical measures in heterogenous patient populations [31, 32]. Notably, our analysis controlled for the use of dexamethasone, a clinical factor known to influence white blood cell count and patient outcomes in GBM [33, 34]. This additional factor may partially explain the observed discrepancy. Importantly, however, many of the underlying trends from the animal models in this highly controlled setting held true.

30. Conflict of interest: a previous publication noted that 'J.R.C is a founder and chairman of the board of Siderobioscience LLC a company founded on patented technology for management of iron deficiency'. Should this be noted?

We did not consider this a conflict of interest, but in the interest of transparency we have added the following to the manuscript:

"James R. Connor is the founder and chairman of the board of Sidero Biosciences LLC, a company with a patented product involving utilization of ferritin for management of iron deficiency. All other authors report no conflicts of interest."

REFEREE 2:

This study presents a valuable contribution to the understanding of iron metabolism in GBM, particularly in highlighting sex-specific responses to systemic iron availability. This manuscript combines an immune-competent GL261 intracranial mouse model with analysis of human GBM samples to test whether dietary systemic iron affects tumor/brain iron handling and survival in a sex-dependent manner. he preclinical work is robust and well-executed, and the integration of human data adds translational context. However, the clinical findings should be interpreted as preliminary due to limited sample size and lack of statistical significance. Some of my concerns are indicated below:

1. **In preclinical results, many comparisons are based on small group sizes, and several key findings hover near conventional significance thresholds. This raises a risk of type-II and type-I errors and of over-interpreting trends. Please report group-wise N for every panel and include confidence intervals and effect sizes, and consider a pre-specified multiplicity correction (or explicitly justify the testing strategy).**

In response to the reviewer, we have included a supplementary table with all of the statistical tests run and the corresponding results (S1 and S2 Tables). Additionally, we have added relevant statistical information into the results and figure legends sections.

2. **Authors state that mice were "arbitrarily placed" on diets at PND21, which is not the same as randomized allocation; IACUC approval is stated, but the manuscript should explicitly describe randomization and whether experimenters were blinded to diet when assessing for example MRI/survival/analyses. Add explicit statements on randomization, allocation concealment, and blinding.**

We have revised the methods section in response to the reviewer to clarify the randomization method as randomized allocation.
We have also stated that investigators performing MRI acquisition/analyses and iron estimation (ICP) analyses were blinded to diet group. The details have been added to the Methods section.

Section 2.1: Mice were placed in group assignments by an individual not involved in downstream data collection. The groups were: control (n = 35; 35 mg/kg Fe, AIN-93G, Inotiv, Cat. #TD.94045) and iron deficient (n = 17; 3.5 mg/kg Fe, #AIN-93, Inotiv, Cat. #TD.10210),) diets at post-natal day (PND) 21.

Section 2.4: Investigators performing MRI acquisition and downstream analyses were blinded to diet group during data collection and analysis.

Section 2.6: Investigators performing ICP measurements were blinded to diet group during data collection and analysis.

3. **In survival analyses, please include multivariable Cox models with interaction terms (sex × iron status, sex × tumor iron) and present HRs with CIs; the Methods mention multivariate Cox models but results are sparse, expand the tables/figures and provide model diagnostic.**

In response to the reviewer, we have replaced the Kaplan-Meier curves in the main figures with the cox proportional hazards regression. The results/figure legends have been updated to represent these results. HRs and CIs can be found within the results, on the graphs within the figures, and in S2 Table. The Kaplan-Meier curves have been relocated to Supplementary Figures 2-4.

4. **For ICP measurements and ELISAs provide detection limits, intra/inter-assay CVs, and whether values below LOD occurred; ensure units are consistently reported because some snippets show g/tissue weight and g/mL.**

Detection limits and statement on LOD was added to the methods and results. For ICP units, g/mL was utilized to express the grams of iron per mL of solution (used only for serum). For clarification, we have gone back to the manuscript to make sure it was clear. Grams of iron/tissue weight was used for tumor and brain tissues, where the weight of the tissue was accounted for in the analysis and used for normalization as described in the methods section.
Sections 2.6 and 2.9 have been substantially edited and the revisions reflect these changes.

5. **The finding that iron-deficient females exhibit increased tumor iron content together with elevated TfR1 and FTH expression is biologically compelling; however, the underlying mechanism remains unclear, as the authors also acknowledge. In its current form, the study does not allow discrimination between alternative, non-mutually exclusive explanations, such as enhanced tumor iron import, tumor cell–intrinsic metabolic adaptations, or redistribution of iron within the tumor microenvironment (for example, accumulation in immune cells with high ferritin content). To strengthen this aspect, the authors should either include additional experiments or more explicitly frame these interpretations as hypotheses for future work. Potential approaches that would help resolve this issue include: (i) iron histochemistry (e.g., Perls' staining) combined with ferritin immunohistochemistry and cell-type–specific markers, (ii) assessment of systemic iron regulators such as transferrin saturation and hepcidin, (iii) cell-type–resolved iron quantification (e.g., flow cytometry, laser-capture microdissection, or spatial transcriptomics), and/or (iv) evaluation of blood–brain barrier permeability.**

We thank the reviewer for this thoughtful comment and agree that the precise mechanisms underlying our findings cannot be definitively resolved within the scope of our current study. Our intent in discussing these possibilities was to frame plausible hypotheses that could guide future mechanistic studies. We believe that discussing the non-mutually exclusive potential explanations provides very valuable framework for further resolution of iron handling in GBM. Per the reviewer's recommendation, we have revised the text to discuss the potential distributions of iron within the tumor and its impact on tumor growth and patient survival and frame these as future work:

The revised manuscript now reads:
Discussion: Therefore, while the changes in protein expression observed here support altered tumor iron handling in IDA females, discerning the mechanisms, including enhanced tumor iron import, specific metabolic adaptations, or redistribution between these diverse cell types, will require future work more specific to cell-specific resolution.
